# Particle shape does not affect ingestion and egestion of microplastics by the freshwater shrimp *Neocaridina palmata*

**DOI:** 10.1007/s11356-021-15068-x

**Published:** 2021-06-29

**Authors:** Kristina Klein, Sebastian Heß, Sandra Nungeß, Ulrike Schulte-Oehlmann, Jörg Oehlmann

**Affiliations:** Faculty of Biological Sciences, Department Aquatic Ecotoxicology, Goethe University Frankfurt, Max-von-Laue-Straße 13, 60438 Frankfurt am Main, Germany

**Keywords:** Polymer, Microplastic, Uptake, Excretion, Freshwater invertebrate, Crustacea, *Neocaridina palmata*

## Abstract

The ingestion of microplastics (MPs) is well documented for various animals and spherical MPs (beads) in many studies. However, the retention time and egestion of MPs have been examined less, especially for irregular MPs (fragments) which are predominantly found in the environment. Furthermore, the accumulation of such particles in the gastrointestinal tract is likely to determine whether adverse effects are induced. To address this, we investigated if the ingestion and egestion of beads are different to those of fragments in the freshwater shrimp *Neocaridina palmata*. Therefore, organisms were exposed to 20–20,000 particles L^−1^ of either polyethylene (PE) beads (41 μm and 87 μm) or polyvinyl chloride (PVC) fragments (<63 μm). Moreover, shrimps were exposed to 20,000 particles L^−1^ of either 41 μm PE and 11 μm polystyrene (PS) beads or the PVC fragments for 24 h, followed by a post-exposure period of 4 h to analyze the excretion of particles. To simulate natural conditions, an additional fragment ingestion study was performed in the presence of food. After each treatment, the shrimps were analyzed for retained or excreted particles. Our results demonstrate that the ingestion of beads and fragments were concentration-dependent. Shrimps egested 59% of beads and 18% of fragments within 4 h. Particle shape did not significantly affect MP ingestion or egestion, but size was a relevant factor. Medium- and small-sized beads were frequently ingested. Furthermore, fragment uptake decreased slightly when co-exposed to food, but was not significantly different to the treatments without food. Finally, the investigations highlight that the assessment of ingestion and egestion rates can help to clarify whether MPs remain in specific organisms and, thereby, become a potential health threat.

**Supplementary Information:**

The online version contains supplementary material available at 10.1007/s11356-021-15068-x.

## Introduction

The ingestion of microplastics (MPs) has been previously described for more than 70 freshwater organisms (summarized by Scherer et al. 2018). With regard to egestion, a comparatively small number of publications are available (Burns and Boxall 2018), focusing on the investigation of either spherical MPs (beads), irregularly shaped MPs (fragments), and fibers or a combination thereof (Au et al. 2015; Blarer and Burkhardt-Holm 2016; Frydkjær et al. 2017; Scherer et al. 2017; Straub et al. 2017; Canniff and Hoang 2018; Weber et al. 2018; Hoang and Felix-Kim 2020). However, the ingestion and egestion capabilities of animals are both important aspects that contribute to potential adverse effects (Fueser et al. 2020), because the residence time of MPs in the digestive system probably determines the level of toxicity (Anbumani and Kakkar 2018). Particle shape could be a relevant factor on handling and passaging time (Frydkjær et al. 2017; Gray and Weinstein 2017) as well as on the relative toxicity. Therefore, it is of particular interest whether rounded beads or sharp-edged fragments need more time to pass the gastrointestinal tract (de Ruijter et al. 2020). After all, comprehensive data on the consumption and elimination of MPs are still lacking for freshwater organisms (Hoang and Felix-Kim 2020).

To address these aspects, we used the freshwater invertebrate *Neocaridina palmata* (var. White Pearl). This shrimp is characterized by a transparent exoskeleton and, therefore, eggs in breeding females, and food uptake is easy to detect. The genus is native to Asia (Karge and Klotz 2013) and typically used there as a model organism in ecotoxicology (EPA/ROC 2013) due to its wide distribution in lakes, streams and ponds (de Grave et al. 2008; Karge and Klotz 2013; Kohal et al. 2018), adaptation to diverse water parameters, relatively short reproduction period and sensitivity to endocrine disrupting chemicals (Huang et al. 2006; Mykles et al. 2016; Huang et al. 2020). Besides, the freshwater organism is increasingly used to address questions relating to decapod physiology (Sonakowska et al. 2015, 2016; Włodarczyk et al. 2017) and genomics (Mykles and Hui 2015; Mykles et al. 2016). Today, it has been found in European rivers (Klotz et al. 2013; Jabłońska et al. 2018), most likely as a result of global trade as an exotic species for hobby aquarists and the unintentional release into the aquatic environment (Schoolmann and Arndt 2018; Jaskuła et al. 2019). We deployed *Neocaridina* as a surrogate organism for decapods in order to approach approximate values for the ingestion of MPs by higher crustaceans such as the endangered noble crayfish *Astacus astacus* (Hilber et al. 2020). We expected that the epibenthic shrimp ingests settled MPs (Haegerbaeumer et al. 2019) and thus incorporated concentrations that cover recently presented data on MPs in the sediment phase (i.e., converted to volumetric units for comparative purposes: 0.51 to 64,900 MPs L^−1^) of global rivers (Scherer et al. 2020). In detail, we investigated the ingestion rate for two differently shaped MPs (i.e., beads and fragments). We further analyzed the retained number of particles in the gut and the egested particles 4 h after the stop of exposure. Finally, we examined whether food interferes with the uptake of fragments, since animals could encounter such particles along with food under environmental conditions.

## Material and methods

### Test organism

*Neocaridina palmata* (var. White Pearl) was purchased and cultured in 20 L glass aquaria at Goethe University (Department Aquatic Ecotoxicology). Individuals were acclimatized at least for 1 week and were kept under constant conditions at 23 ± 2 °C and a 16:8 h light/dark cycle (460 lux). Reconstituted water based on the *OECD guideline 242*: *Potamopyrgus antipodarum Reproduction Test* (OECD 2016) was used in diluted form (i.e., 60%) to obtain a pH of 7.5 ± 1.0 and conductivity of 400 ± 100 μS cm^−1^. Therefore, 1.8 g Tropic Marin® sea salt and 1.08 g NaHCO_3_ were dissolved per 10 L of deionized water. The aquaria were provided with nano corner filters (Dennerle GmbH, Münchweiler an der Rodalb, Germany) and continuous aeration. Twice a week, the medium was partially renewed, and the shrimps were fed *ad libitum* with CrustaGran and Shrimp King Mineral (Dennerle GmbH). The number of individuals in the culturing aquaria varied greatly, depending on the reproduction rate of the individuals at the time.

### Test materials

Spherical MPs and fluorescent polyethylene (PE) beads (excitation maximum: 414 nm, emission maximum: 515 nm) of two different size ranges (UVPMS-BG-1.035g/cc 38–45 μm and UVPMS-BG-1.025g/cc 75–90 μm) were purchased from Cospheric LLC© (Santa Barbara, USA) and Fluoresbrite® YG 10 μm polystyrene (PS) beads in a 2.5% aqueous suspension (article no. 18140, excitation: 441 nm, emission: 486 nm) from Polysciences Europe GmbH (Hirschberg an der Bergstrasse, Germany). Irregular MPs (fragments) were prepared from a fluorescent (excitation: 400–410 nm, emission: 455 nm) polyvinyl chloride (PVC) cord (Modulor GmbH, Berlin, Germany); the PVC cord was cut into small pieces (<1 cm) and milled cryogenically for 1–2 min at 30 Hz (Mixer Mill MM400, Retsch GmbH, Haan, Germany). The grinding steps were repeated until a fine powder was formed, which was sieved (<63 μm) using the Vibratory Sieve Shaker AS 200 basic (Retsch GmbH, Haan, Germany). Since there were no data available for the specific density of the PVC cord, the density was determined based on the weight and volume of one PVC cord piece (Table 1). The average size of each MP was determined by measuring 100 beads and 150 fragments with the Olympus BX50 fluorescence microscope and a connected digital camera (JVC KY-F75U and Olympus UC90). PVC fragments ≤5 μm were generally not considered for analysis due to optical limitations (Table 1, Fig. S1 and Fig. S2). Since the PVC fragments comprised irregular forms, the surface structure of these particles was analyzed with the S-4500 Hitachi Scanning Electron Microscope (Fig. S3).

**Table 1:** Properties of beads and fragments used in the ingestion and egestion studies

Experiment	Ingestion study			Egestion study		
	Beads^a^		Fragments	Beads^a^		Fragments
Polymer type	PE	PE	PVC	PE	PS	PVC
Density [g cm^−3^]	1.03	1.04	1.26	1.04	1.05	1.26
Mean size ± SD [μm]	87.0 ± 4.83	41.1 ± 3.42	22.0 ± 16.8	41.1 ± 3.42	11.5 ± 0.87	22.0 ± 16.8

^a^Exposed as mixtures (1:1)

Each bead type was suspended with ultrapure water and the surfactant Tween®20 (CAS 9005-64-5, Sigma-Aldrich) to avoid the agglomeration of beads (Frydkjær et al. 2017), not exceeding a final solvent concentration of 0.01% (v/v). Stock suspensions of fragments were prepared directly with medium (Table S1). Stock suspensions with beads were shaken for 24 h at 120 rpm, while 300 rpm were necessary to disperse the fragments (GFL 3017, Burgwedel, Germany). In order to determine the particle concentration of each suspension, aliquots were taken and vacuum-filtered onto cellulose nitrate membrane filters of 0.8 μm pore size (Sartorius AG, Göttingen, Germany). Retained particles were optically counted using the fluorescence microscope; particles ≤5 μm were not considered. Based on the derived concentrations, volumes from the stock suspensions, corresponding to the test concentrations, were rechecked to ensure that nominal and actual particle concentrations matched (see Table S1). Subsequently, the appropriate volumes were added to the test vessels. All vessels were prepared at least 20 h before the addition of the shrimps and remained without aeration to allow MP settlement. As the physical properties of the examined MPs differed (Table 1), we analyzed the agglomeration behavior of the particles. Due to their bright coloring, we could observe that beads accumulated on the bottom of the test vessels. Since fragments were not fully visible to the eye, the fragment settlement was investigated further (Fig. S4). Settlement of the fragments was confirmed after 20 h and remained at a similar level when the test vessels were aerated for an additional 24-h period (Fig. S4). The latter resembled the actual exposure conditions for 24 h.

### Ingestion and egestion studies

Prior to the experiments, adult organisms were selected by size and allocated to other tanks that included the minimum number of adults needed for each experiment. The individuals were then held for 24 h in vessels with particle-free medium to allow gut clearance; all tested individuals had a mean body length of 12.7 ± 1.48 mm (Table S3). All treatments had eight replicates, with one individual per vessel and 500 mL medium, respectively, and were conducted once. In order to prevent the resuspension of particles, the test vessels were aerated a few centimeters below the surface of the medium for the test period. At the beginning and end of all tests, water parameters (pH, conductivity, oxygen, and temperature) were measured (Table S2).

For the ingestion study, individuals were exposed to four concentrations of beads and fragments (20, 200, 2000, and 20,000 particles L^−1^) for 24 h (Table 1), respectively. These concentrations mirror global concentrations of MPs in sediments of rivers (Scherer et al. 2020). We expected the shrimps to encounter such MPs since the epibenthic organism feeds on biofilm material on the substrate (Pantaleão et al. 2017). We chose an exposure period of 24 h in order to reach a steady MP buildup (Rist et al. 2017). Negative controls without MPs were conducted in parallel. Since Pikuda et al. (2019) demonstrated that surfactants can negatively impact *Daphnia magna*, we included a solvent control with 0.01% (v/v) of Tween®20 as we dispersed the beads with this solution. To elucidate whether the shrimps feed preferentially within a specific size range, the experiments with beads were conducted with mixtures (1:1) of 75–90 μm and 38–45 μm PE beads in the ingestion study and 38–45 μm PE and 10 μm PS beads in the egestion study, respectively (Table 1). In addition to the ingestion study with MP fragments <63 μm, the effect of available food on the ingestion of fragments was investigated. Thus, *N. palmata* was exposed to similar treatments (20, 200, 2000, and 20,000 fragments L^−1^) for 24 h but with added 4–5 mg of CrustaGran per test vessel; this food quantity corresponds approximately to 10% of the shrimps’ wet weight (Vazquez et al. 2017). In addition, food was added to the negative control in order to detect potential synthetic particles introduced by the food source itself (Table S4). The food was added once and settled to the bottom of the test vessels.

To determine the number of ingested particles from the aforementioned experiments, individuals were rinsed with ultrapure water at the end of the exposure period to ensure the complete runoff of attached particles, snap-frozen in liquid nitrogen, and stored at −20 °C until further analysis. The body length (defined as the distance from the rostrum to the posterior margin of the last abdominal segment) and the sex (by means of the *appendix masculina*) were determined for each individual using an Olympus SZ40 stereo microscope (Table S3). Animals were again rinsed with ultrapure water and lysed in a 1:10 solution of 10% H_2_SO_4_ and 30% H_2_O_2_ for 72 h (40 °C, 300 rpm) (Heidolph Titramax 1000 with Inkubator 1000, Heidolph Instruments GmbH & Co. KG, Schwabach, Germany). Lysates were then vacuum-filtered onto cellulose nitrate membrane filters and analyzed for ingested particles using the fluorescence microscope. The data were corrected for the negative control of the beads that served as a blank and for the airborne control that was necessary during the microscopical fragment analysis (Table S4).

For the egestion study, 16 shrimps were exposed for 24 h to the highest concentration (20,000 particles L^−1^) of a PE-PS beads mixture and PVC fragments, respectively. Half of the individuals were then transferred into particle-free vessels with food (10 mg CrustaGran), which was added once to the vessels. A higher food amount than in the fragment ingestion study with food was chosen to increase the encounter rate for natural particles and, thereby, enhance the excretion. A post-exposure period of 4 h (t = 4 h) for the egestion of particles was chosen since preliminary tests revealed that <4 h is sufficient for the shrimps to egest more than 50% of beads. The other half of the individuals not intended for excretion analysis were removed from the test after particle exposure to serve as a reference for particle uptake (t = 0 h). After the egestion period, the shrimps were cleaned and lysed under the same conditions as previously described. Lysates and excretions were vacuum-filtered and analyzed microscopically next to the shrimps that had no egestion period (t = 0 h). Here, the negative control of the beads study served as a blank, while another filter accounted for the introduction of airborne fragment-like particles during microscopy. A further blank accounted for potential fragment-like particles introduced by the food source during post-exposure (Table S4).

### Data analysis

Data were analyzed with GraphPad Prism® (5.00 and 9.00) (GraphPad Software Inc., San Diego, USA). The data were tested for normal distribution. If the data were not normally distributed or in cases of variance inhomogeneity, the Kruskal-Wallis test followed by Dunn’s post hoc test was conducted; otherwise a one-way ANOVA with Dunnett’s post hoc test was performed. Statistical comparisons were made between the control group without MPs and the exposure treatments. Relationships between the body length and ingested or egested particles were analyzed using the Pearson or Spearman correlations, depending on whether the data met the parametric criteria. In order to test if the particle type, sex, and added food influenced the ingestion or egestion, a two-way ANOVA with Bonferroni’s post hoc test was performed. The significance level was defined with α = 0.05 (*p* <0.05, *p* <0.01, *p* <0.001, and *p* <0.0001).

## Results

### Ingestion study

**Fig. 1:**
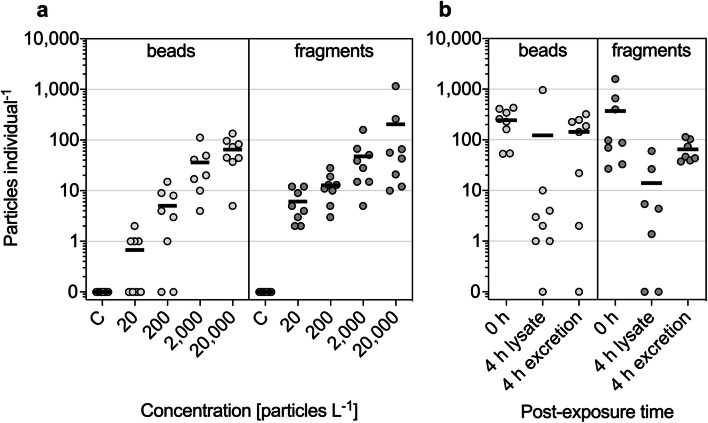
*Neocaridina palmata*. **a** Mean number (lines) of detected beads and fragments individual^−1^ in shrimp lysates for the ingestion study. **b** Mean number (lines) of detected beads and fragments in shrimp lysates exposed to 20,000 particles L^−1^ (t = 0 h), as well as in the shrimp lysates and their corresponding excretions after an additional egestion period of 4 h (t = 4 h). No beads or fragments were added to the controls (C) in the ingestion study. One independent experiment with *n* = 7–8 replicates for each treatment

*Neocaridina palmata* ingested both beads and fragments in a concentration-dependent manner (Fig. 1a). The respective negative controls, including the solvent control for the bead testing, contained neither beads nor PVC-like particles. It was necessary to correct the fragment data since one PVC-like fragment was detected in the airborne blank (Table S4). In general, the number of PE beads found in the lysates increased (0.63–64.6 beads individual^−1^) with rising exposure concentrations (20–20,000 beads L^−1^) (Table S4), whereas mostly beads of the smaller size class (38–45 μm) were detected compared to the 75–90 μm beads. Regarding the 20,000 beads L^−1^ exposure treatment, for instance, shrimps ingested 60.8 beads of the 38–45 μm size class and 3.80 beads of the 75–90 μm size class. Compared to the control without MPs, significant increases were observed for the exposure to 2000 (*p* <0.001) and 20,000 (*p* <0.0001) beads L^−1^. During exposure to 2000 beads L^−1^, one individual out of eight individuals died. Regarding the PVC particles, the mean number of ingested particles ranged from 6.13 to 204 fragments individual^−1^ after exposure to 20–20,000 fragments L^−1^. Once again, significant increases were observed for 200 fragments L^−1^ (*p* <0.01) as well as for 2000 and 20,000 fragments L^−1^ (*p* <0.0001) when compared to the control group. A significant influence of the differently shaped MPs in relation to the uptake was not detected. Moreover, neither significant differences for the MP ingestion between males and females nor a correlation between MP ingestion and body length were observed (Table S3).

### Egestion study

Based on the results of the ingestion study (i.e., shrimps ingested a higher number of the smaller sized PE beads (38–45 μm) compared to the 75–90 μm PE beads), the organisms were further exposed to a PE-PS beads mixture of an even smaller size range in the egestion experiment (Table 1). For the egestion experiment (Fig. 1b), a reference (t = 0 h) was carried out for the ingestion of 38–45 μm PE and 10 μm PS beads and the <63 μm PVC fragments for shrimps exposed to 20,000 particles L^−1^. In another treatment, individuals had an additional post-exposure time (t = 4 h) in particle-free medium to allow the measurement of egested particles. Here, the shrimps’ excretions as well as lysates were examined to elucidate whether particles remained in the digestive system. On average, the shrimps contained 243 beads individual^−1^ (t = 0 h), i.e., 146 of 11 μm beads and 96.4 of 41 μm beads, and egested 143 beads individual^−1^ after 4 h (i.e., 59% of the previously ingested beads); the latter was corrected for two beads found in the corresponding negative control (Table S4). After 4 h of post-exposure time, 123 beads individual^−1^ remained in the shrimp lysates but were still significantly different to the reference treatment (*p* <0.05) (Table S4, Fig. 1b). *Neocaridina palmata* was further observed to excrete irregularly shaped MPs. In the excretions, 65.1 PVC fragments individual^−1^ were detected, while the food itself introduced 1.63 PVC-like particles (Table S4). The mean number of fragments individual^−1^ decreased significantly (*p* <0.01) from 371 in the reference (t = 0 h) to 14.0 in the lysate within 4 h of post-exposure. One individual died in the egestion treatment (t = 4 h) (Table S4, Fig. 1b). No significant difference between the egestion of beads and fragments was observed. Furthermore, no correlations between the body length and egestion or sex-specific differences could be detected. Due to the high variability that could potentially mask effects, the ingestion and egestion data were corrected for statistical outliers (Grubb’s test) and evaluated again. This data resulted in similar findings as already described.

### Food availability

**Fig. 2:**
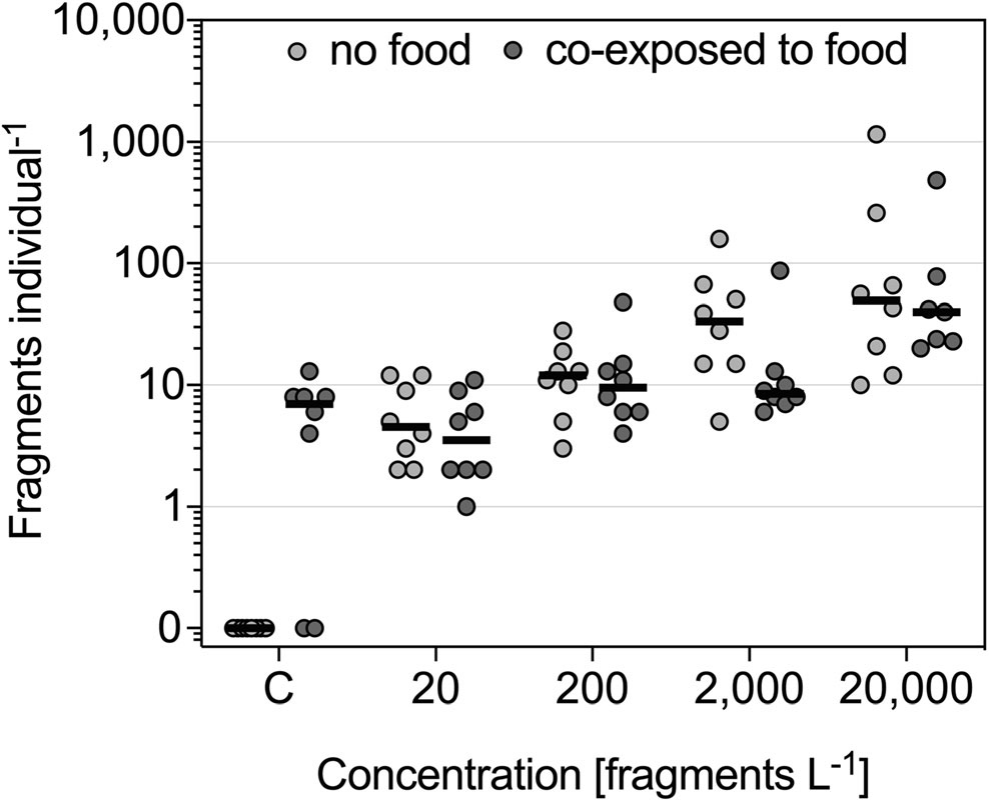
*Neocaridina palmata*. Mean number (lines) of detected fragments individual^−1^ in shrimp lysates exposed to PVC fragments in the absence and presence of food. No fragments were added to the controls (C), but the food source introduced PVC-like fragments. One independent experiment with *n* = 7–8 replicates for each treatment

Finally, we investigated whether food availability influenced the ingestion of fragments (Fig. 2). The ingested fragments without food resemble the same data as illustrated in Fig. 1a. The negative control with food contained 5.88 PVC-like particles individual^−1^ and, therefore, included more particles than the exposure treatment with 20 fragments L^−1^. Here, an average of 4.75 PVC particles was detected per shrimp (Fig. 2, Table S4). The setup demonstrated similar ingestion rates as in the experiment without food, but with slightly lower mean ingested particles individual^−1^ for the two highest treatments. However, no significant difference was found between the treatments in the presence and absence of food. During the exposure to 20,000 fragments L^−1^, one individual died. Overall, mortality occurred for one individual each in the ingestion experiment exposed to 2000 beads L^−1^, co-exposed to 20,000 fragments L^−1^ and food as well as in the fragment egestion experiment following the 4 h excretion period (t = 4 h).

## Discussion

### Ingestion rates of beads and fragments are comparable

The current study aimed to examine differences in the gut passaging for microplastic beads and fragments by the atyid shrimp *Neocaridina palmata*. In addition, we used MP concentrations measured in the sediment of global freshwaters (Scherer et al. 2020). The ingestion for both MP shapes was concentration-dependent (Fig. 1a). Based on the ingestion and egestion (t = 0 h) study, shrimps frequently ingested medium- and small-sized beads (i.e., 41 μm PE and 11 μm PS beads, respectively) compared to the large-sized beads of the respective exposure scenario (Fig. 1a, b). Thus, we detected size-related uptake preferences. However, the ingestion of both MP shapes did not differ significantly. In contrast, the estuarine shrimp *Palaemonetes pugio* was observed to ingest significantly higher numbers of 34 μm and 93 μm polypropylene (PP) fragments than of 30–165 μm PE and PS beads (Gray and Weinstein 2017); this could indicate shape-related influences. Lehtiniemi et al. (2018) somewhat support this as the mysid shrimp *Mysis relicta* ingested high rates of acrylonitrile butadiene styrene (ABS) fragments, but not polyethylene terephthalate (PET) fragments, when compared to PS beads. This could be attributed to the individual’s ingestible size range since ABS fragments were smaller than the PET. Moreover, diverse MP properties (e.g., size, density, and surface chemistry) could contribute to bioavailability issues (Lambert et al. 2017). For instance, Frydkjær et al. (2017) showed that fragment uptake can decrease in daphnids, despite rising concentrations, when MPs agglomerate and are out of reach. Although the physical properties of our MPs differed (see Table 1), we detected that beads and fragments sedimented (Fig. S4) and so both were similarly available to the shrimps; this is in line with Setälä et al. (2016). They examined PS beads as used in the present study and observed them to settle, thereby becoming available for ingestion by the mysid shrimps *Neomysis integer* and *Praunus flexuosus*. Therefore, we do not assume that the sedimentation had a major impact on the study results. Considering the preferential uptake of the lower sized beads, it could be argued that the PVC fragments with the broad dimension range have been ingested disproportionally compared to the spheres with tight size specifications (Table 1). However, we generally excluded the lowest size range of the fragments (i.e., ≤5 μm) and, thereby, disregarded at least the smallest MPs. In general, our results indicate a rather unselective ingestion of MPs by *N. palmata*, which is likely connected to its opportunistic omnivorous feeding strategy (Yam and Dudgeon 2005; Weber and Traunspurger 2016). Thus, it is not surprising that the ingestion of beads and fragments were not significantly different.

### Fragment uptake tends to be lower in the presence of food

We investigated the ingestion of fragments while food was available to the shrimps. We could not detect significant differences between the fragment ingestion in the absence and presence of food but observed a tendency towards a slightly reduced fragment uptake for individuals co-exposed to food (Fig. 2). Along this line, other freshwater invertebrates such as *D. magna* and *Gammarus pulex* have been shown to have reduced uptake rates for MPs in the presence of algae or leaf material (Scherer et al. 2017; Aljaibachi and Callaghan 2018). Bour et al. (2020) reported that the brine shrimp *Artemia* ingested less PE beads when co-exposed to food. However, the feeding type of these animals is not the same as for *Neocaridina*. Recent studies have described different outcomes when focusing on caridean shrimps as used in the present study. For instance, Saborowski et al. (2019) examined the uptake of polyacrylic wool fibers and different food concentrations with the Atlantic ditch shrimp *Palaemon varians*. In the cases where commercial food was present compared to when exposure took place without food, they demonstrated that the number of ingested microfibers was higher. This was explained by fibers attaching to the food source and, thereby, facilitating ingestion. They observed regurgitation of large microfibers via the esophagus of *P. varians*, highlighting the ability to remove indigestible particles. Korez et al. (2020) found PS beads in the stomach and midgut gland of the brown shrimp *Crangon crangon*, but, due to this organism being a predator, they generally included food to increase particle interaction. Therefore, it cannot be distinguished whether the beads would be ingested to a higher or lower extent in the absence of food by the brown shrimp. However, they examined the ingestion of inorganic particles (e.g., quartz grains and fragments from the remains of bivalve shells) and detected high loads of natural particles. This indicated active particle uptake enabling food to be mechanically fragmented. Based on this observation, they concluded that shrimps may be less selective in their search for food and therefore less susceptible towards MP contamination in their environment. Our findings are plausible in that food probably reduces the animals’ encounter rate for MPs due to dilution effects (summarized by de Ruijter et al. 2020). However, it does not seem necessarily relevant for the epibenthic shrimp whether food is present or not because they likely feed on various sediment constituents. We argue that *N. palmata* does not appear to selectively feed on certain particles; this agrees with its omnivorous feeding behavior.

### Comparably fast excretions of beads and fragments

Our egestion experiments demonstrated that *N. palmata* can excrete previously ingested MPs (i.e., t = 0 h) within 4 h of post-exposure. The shrimps only partially egested the particles within this specific excretion period as 123 beads and 14 fragments remained in the gastrointestinal tract (Fig. 1b). Interestingly, we did not observe a statistical difference between the egestion of beads and fragments. Similarly, Gray and Weinstein (2017) tested the egestion of 11 different MPs with the estuarine shrimp *P. pugio* and observed no apparent trend towards a prolonged residence time of differently sized as well as shaped MPs. Likewise, the same species egested the majority of ingested PE spheres and PP fragments within 2 days (Leads et al. 2019). Korez et al. (2020) demonstrated that *C. crangon* egested the majority of PS beads after 24 to 48 h. However, they could not exclude the reentrance of MPs from feces due to coprophagy. The same may be relevant for our study since we found some MPs in the shrimps’ lysates (Fig. 2b) during the post-exposure time. In order to conclude about incomplete excretion, the excretion time should be longer and the experimental design must monitor the excretion over time without allowing the organisms to re-ingest excreted particles. After all, the egestion of MPs is crucial in terms of limited gut space for the consumption of real nutritious food, which could result in energy depletion and developmental delays (Hoang and Felix-Kim 2020). We selected 4 h as the post-exposure period based on a preliminary conducted egestion study (data not shown) for beads at different times (4, 8, 16, and 32 h). Here, we could not detect significant differences between the excretion groups. Our data indicate that 59% of beads were excreted after 4 h (Fig. 2b). However, when we combined the groups of different excretion times from the preliminary test to obtain a large dataset (*n* = 32 replicates), we observed a comparably higher excretion rate for beads (85%), while only a small fraction was found in the digestive systems, and the rest could not be detected due to methodological reasons. Saborowski et al. (2019) demonstrated that the stomachs of *P. varians* were emptied from beads and fibers after 16–24 h. Bour et al. (2020) support this observation since they demonstrated major and complete bead depuration in *Artemia* after 24 and 48 h, respectively. Leads et al. (2019) showed that the egestion of different MP shapes is not affected in shrimps, which were previously injected with the bacterium *Vibrio campbellii* to increase their susceptibility to MPs. Taken together, our results are mostly in line with other publications and highlight that beads as well as fragments pass the shrimp’s gut. Due to the numerous aspects that can influence the ingestion and egestion of MPs, a transfer of our results to other species (e.g., crayfish as higher crustaceans) is very limited, and solely the analysis of sampled animals would elucidate true accumulation rates of MPs (comp. Zhang et al. 2020).

It is noteworthy that three individuals died, which was however not exclusive to one MP shape. Canniff and Hoang (2018), for instance, used high concentrations of up to 100 mg L^−1^ of similar PE beads and did not detect adverse effects on the survival of *D. magna*. Cytotoxic effects could not either be detected in *in vitro* models with human cell lines (Çobanoğlu et al. 2021; Stock et al. 2021), except at really high concentrations (i.e., >75 mg mL^−1^) for PE beads and powdered PVC particles by Stock et al. (2021). Given the comparably low MP concentrations examined in the present study, we cannot ascribe a specific toxicity mechanism to the low mortality of *Neocaridina*. In order to elucidate the real cause for the mortality, further research has to be performed with specific regard to internal injuries due to sharp-edged fragments or migrating chemicals from MPs.

## Conclusions

We exposed *Neocaridina palmata* to realistic MP concentrations measured in the sediment of freshwaters and showed that shrimps generally ingest MPs. We further demonstrated that both the ingestion and egestion of beads and fragments do not differ in the freshwater organism. The particle size but not the shape affected the uptake. Moreover, we did not detect any significant differences between the fragment ingestion in the presence and absence of food, but we observed a slight tendency towards lower fragment uptake with the availability of food. This could reflect environmental conditions. Taken together, we could not detect any influencing factors on the ingestion other than the individuals’ mouth opening probably limiting the ingestible particle size. Our results indicate that *Neocaridina* is not very selective regarding food properties, which might be linked to its omnivorous feeding behavior. We further observed that shrimps rapidly but only partially egested beads and fragments within 4 h. As the depuration was incomplete within this time frame, long-term effects cannot be fully excluded based on our study. Moreover, it is not reasonable to ascribe the low observed mortality rate to a specific toxicity mechanism, considering the low MP concentrations used. However, since we mostly observed few remaining particles in the digestive tract and shrimps are known to ingest high natural particle loads, we assume that the physical impact of MPs would be minor for freshwater shrimps. Overall, we are convinced that the assessment of ingestion and egestion rates is an important preliminary step for chronic studies. This could generally help to clarify whether MPs accumulate in organisms and, thereby, become a potential health problem at the individual level or even for higher animals via trophic transfer.

## Supplementary Information

ESM 1 (DOCX 4720 kb)

## Data Availability

The data used in the present study are available from the corresponding author on reasonable request.

## References

[CR1] Aljaibachi R, Callaghan A (2018). Impact of polystyrene microplastics on *Daphnia magna* mortality and reproduction in relation to food availability. PeerJ.

[CR2] Anbumani S, Kakkar P (2018). Ecotoxicological effects of microplastics on biota: a review. Environ Sci Pollut Res.

[CR3] Au SY, Bruce TF, Bridges WC, Klaine SJ (2015). Responses of *Hyalella azteca* to acute and chronic microplastic exposures. Environ Toxicol Chem.

[CR4] Blarer P, Burkhardt-Holm P (2016). Microplastics affect assimilation efficiency in the freshwater amphipod *Gammarus fossarum*. Environ Sci Pollut Res.

[CR5] Bour A, Hossain S, Taylor M, Sumner M, Almroth BC (2020). Synthetic microfiber and microbead exposure and retention time in model aquatic species under different exposure scenarios. Front Environ Sci.

[CR6] Burns EE, Boxall ABA (2018). Microplastics in the aquatic environment: evidence for or against adverse impacts and major knowledge gaps. Environ Toxicol Chem.

[CR7] Canniff PM, Hoang TC (2018). Microplastic ingestion by *Daphnia magna* and its enhancement on algal growth. Sci Total Environ.

[CR8] Çobanoğlu H, Belivermiş M, Sıkdokur E, Kılıç Ö, Çayır A (2021). Genotoxic and cytotoxic effects of polyethylene microplastics on human peripheral blood lymphocytes. Chemosphere.

[CR9] De Grave S, Cai Y, Anker A (2008). Global diversity of shrimps (Crustacea: Decapoda: Caridea) in freshwater. Hydrobiologia.

[CR10] De Ruijter VN, Redondo-Hasselerharm PE, Gouin T, Koelmans AA (2020). Quality criteria for microplastic effect studies in the context of risk assessment: a critical review. Environ Sci Technol.

[CR11] EPA/ROC (2013) Standard guide for conducting acute tests with shrimps: static renewal test for *Neocaridina denticulata*, NIEA B905.13B. Environmental Protection Administration of Taiwan, Taipei

[CR12] Frydkjær CK, Iversen N, Roslev P (2017). Ingestion and egestion of microplastics by the cladoceran *Daphnia magna*: effects of regular and irregular shaped plastic and sorbed phenanthrene. Bull Environ Contam Toxicol.

[CR13] Fueser H, Mueller M-T, Traunspurger W (2020). Rapid ingestion and egestion of spherical microplastics by bacteria-feeding nematodes. Chemosphere.

[CR14] Gray AD, Weinstein JE (2017). Size- and shape-dependent effects of microplastic particles on adult daggerblade grass shrimp (*Palaemonetes pugio*): uptake and retention of microplastics in grass shrimp. Environ Toxicol Chem.

[CR15] Haegerbaeumer A, Mueller M-T, Fueser H, Traunspurger W (2019). Impacts of micro- and nano-sized plastic particles on benthic invertebrates: a literature review and gap analysis. Front Environ Sci.

[CR16] Hilber T, Oehm J, Effenberger M, Maier G (2020). Evaluating the efficiency of three methods for monitoring of native crayfish in Germany. Limnologica.

[CR17] Hoang TC, Felix-Kim M (2020). Microplastic consumption and excretion by fathead minnows (*Pimephales promelas*): influence of particles size and body shape of fish. Sci Total Environ.

[CR18] Huang C-W, Chu P-Y, Wu Y-F, Chan W-R, Wang Y-H (2020). Identification of functional SSR markers in freshwater ornamental shrimps *Neocaridina denticulata* using transcriptome sequencing. Mar Biotechnol.

[CR19] Huang D-J, Chen H-C, Wu J-P, Wang S-Y (2006). Reproduction obstacles for the female green neon shrimp (*Neocaridina denticulata*) after exposure to chlordane and lindane. Chemosphere.

[CR20] Jabłońska A, Mamos T, Gruszka P, Szlauer-Łukaszewska A, Grabowski M (2018). First record and DNA barcodes of the aquarium shrimp, *Neocaridina davidi*, in Central Europe from thermally polluted River Oder canal, Poland. Knowl Manag Aquat Ecosyst.

[CR21] Jaskuła R, Sulikowska-Drozd A, Jabłońska A, Banaś K, Rewicz T (2019). Undesirable immigrants: hobbyist vivaria as a potential source of alien invertebrate species. PeerJ.

[CR22] Karge A, Klotz W (2013). Süßwassergarnelen aus aller Welt.

[CR23] Klotz W, Miesen FW, Hüllen S, Herder F (2013). Two Asian fresh water shrimp species found in a thermally polluted stream system in North Rhine-Westphalia, Germany. Aquat Invasions.

[CR24] Kohal MN, Fereidouni AE, Firouzbakhsh F, Hayati I (2018) Effects of dietary incorporation of *Arthrospira* (*Spirulina*) *platensis* meal on growth, survival, body composition, and reproductive performance of red cherry shrimp *Neocaridina davidi* (Crustacea, Atyidae) over successive spawnings. J Appl Phycol 30:431-443. 10.1007/s10811-017-1220-5

[CR25] Korez Š, Gutow L, Saborowski R (2020). Coping with the “dirt”: brown shrimp and the microplastic threat. Zool.

[CR26] Lambert S, Scherer C, Wagner M (2017). Ecotoxicity testing of microplastics: considering the heterogeneity of physicochemical properties. Integr Environ Assess Manag.

[CR27] Leads RR, Burnett KG, Weinstein JE (2019). The effect of microplastic ingestion on survival of the grass shrimp *Palaemonetes pugio* (Holthuis, 1949) challenged with *Vibrio campbellii*. Environ Toxicol Chem.

[CR28] Lehtiniemi M, Hartikainen S, Näkki P, Engström-Öst J, Koistinen A, Setälä O (2018). Size matters more than shape: ingestion of primary and secondary microplastics by small predators. Food Webs.

[CR29] Mykles DL, Burnett KG, Durica DS, Joyce BL, McCarthy FM, Schmidt CJ, Stillman JH (2016). Resources and recommendations for using transcriptomics to address grand challenges in comparative biology. Integr Comp Biol.

[CR30] Mykles DL, Hui JHL (2015). *Neocaridina denticulata*: a decapod crustacean model for functional genomics. Integr Comp Biol.

[CR31] OECD (2016) Guidelines for the testing of chemicals - *Potamopyrgus antipodarum* reproduction test. Guideline 242, adopted 29 July 2016. OECD, Paris

[CR32] Pantaleão JAF, Gregati RA, da Costa RC, López-Greco LS, Negreiros-Fransozo ML (2017). Post-hatching development of the ornamental ‘red cherry shrimp’ *Neocaridina davidi* (Bouvier, 1904) (Crustacea, Caridea, Atyidae) under laboratorial conditions. Aquac Res.

[CR33] Pikuda O, Xu EG, Berk D, Tufenkji N (2019). Toxicity assessments of micro- and nanoplastics can be confounded by preservatives in commercial formulations. Environ Sci Technol Lett.

[CR34] Rist S, Baun A, Hartmann NB (2017). Ingestion of micro- and nanoplastics in *Daphnia magna* – quantification of body burdens and assessment of feeding rates and reproduction. Environ Pollut.

[CR35] Saborowski R, Paulischkis E, Gutow L (2019). How to get rid of ingested microplastic fibers? A straightforward approach of the Atlantic ditch shrimp *Palaemon varians*. Environ Pollut.

[CR36] Scherer C, Brennholt N, Reifferscheid G, Wagner M (2017). Feeding type and development drive the ingestion of microplastics by freshwater invertebrates. Sci Rep.

[CR37] Scherer C, Weber A, Lambert S, Wagner M, Wagner M, Lambert S (2018). Interactions of microplastics with freshwater biota. Freshwater microplastics. The handbook of environmental chemistry.

[CR38] Scherer C, Weber A, Stock F, Vurusic S, Egerci H, Kochleus C, Arendt N, Foeldi C, Dierkes G, Wagner M, Brennholt N, Reifferscheid G (2020). Comparative assessment of microplastics in water and sediment of a large European river. Sci Total Environ.

[CR39] Schoolmann G, Arndt H (2018). Population dynamics of the invasive freshwater shrimp *Neocaridina davidi* in the thermally polluted Gillbach stream (North Rhine-Westphalia, Germany). Limnologica.

[CR40] Setälä O, Norkko J, Lehtiniemi M (2016). Feeding type affects microplastic ingestion in a coastal invertebrate community. Mar Pollut Bull.

[CR41] Sonakowska L, Włodarczyk A, Poprawa I, Binkowski M, Śróbka J, Kamińska K, Kszuk-Jendrysik M, Chajec Ł, Zajusz B, Rost-Roszkowska MM (2015). Structure and ultrastructure of the endodermal region of the alimentary tract in the freshwater shrimp *Neocaridina heteropoda* (Crustacea, Malacostraca). PLoS One.

[CR42] Sonakowska L, Włodarczyk A, Wilczek G, Wilczek P, Student S, Rost-Roszkowska MM (2016). Cell death in the epithelia of the intestine and hepatopancreas in *Neocaridina heteropoda* (Crustacea, Malacostraca). PLoS One.

[CR43] Stock V, Laurisch C, Franke J, Dönmez MH, Voss L, Böhmert L, Braeuning A, Sieg H (2021). Uptake and cellular effects of PE, PP, PET and PVC microplastic particles. Toxicol in Vitro.

[CR44] Straub S, Hirsch PE, Burkhardt-Holm P (2017). Biodegradable and petroleum-based microplastics do not differ in their ingestion and excretion but in their biological effects in a freshwater invertebrate *Gammarus fossarum*. Int J Environ Res Public Health.

[CR45] Vazquez ND, Delevati-Colpo K, Sganga DE, López-Greco LS (2017). Density and gender segregation effects in the culture of the caridean ornamental red cherry shrimp *Neocaridina davidi* Bouvier, 1904 (Caridea: Atyidae). J Crustac Biol.

[CR46] Weber A, Scherer C, Brennholt N, Reifferscheid G, Wagner M (2018). PET microplastics do not negatively affect the survival, development, metabolism and feeding activity of the freshwater invertebrate *Gammarus pulex*. Environ Pollut.

[CR47] Weber S, Traunspurger W (2016). Influence of the ornamental red cherry shrimp *Neocaridina davidi* (Bouvier, 1904) on freshwater meiofaunal assemblages. Limnologica.

[CR48] Włodarczyk A, Sonakowska L, Kamińska K, Marchewka A, Wilczek G, Wilczek P, Student S, Rost-Roszkowska MM (2017). The effect of starvation and re-feeding on mitochondrial potential in the midgut of *Neocaridina davidi* (Crustacea, Malacostraca). PLoS One.

[CR49] Yam RSW, Dudgeon D (2005). Stable isotope investigation of food use by *Caridina* spp. (Decapoda:Atyidae) in Hong Kong streams. J N Am Benthol Soc.

[CR50] Zhang D, Fraser MA, Huang W, Ge C, Wang Y, Zhang C, Guo P (2020). Microplastic pollution in water, sediment, and specific tissues of crayfish (*Procambarus clarkii*) within two different breeding modes in Jianli, Hubei province, China. Environ Pollut.

