# Supplementary material for: Particle shape does not affect ingestion and egestion of microplastics by the freshwater shrimp Neocaridina palmata
Source: Environ Sci Pollut Res Int. 2021 Jun 29;28(44):62246–54. doi: 10.1007/s11356-021-15068-x (PMC8589796; doi:10.1007/s11356-021-15068-x)
Supplement: Supplementary file 1 — (DOCX 4720 kb) [file 11356_2021_15068_MOESM1_ESM.docx]

**20 µm**

**200 µm**

**200 µm**

**200 µm**


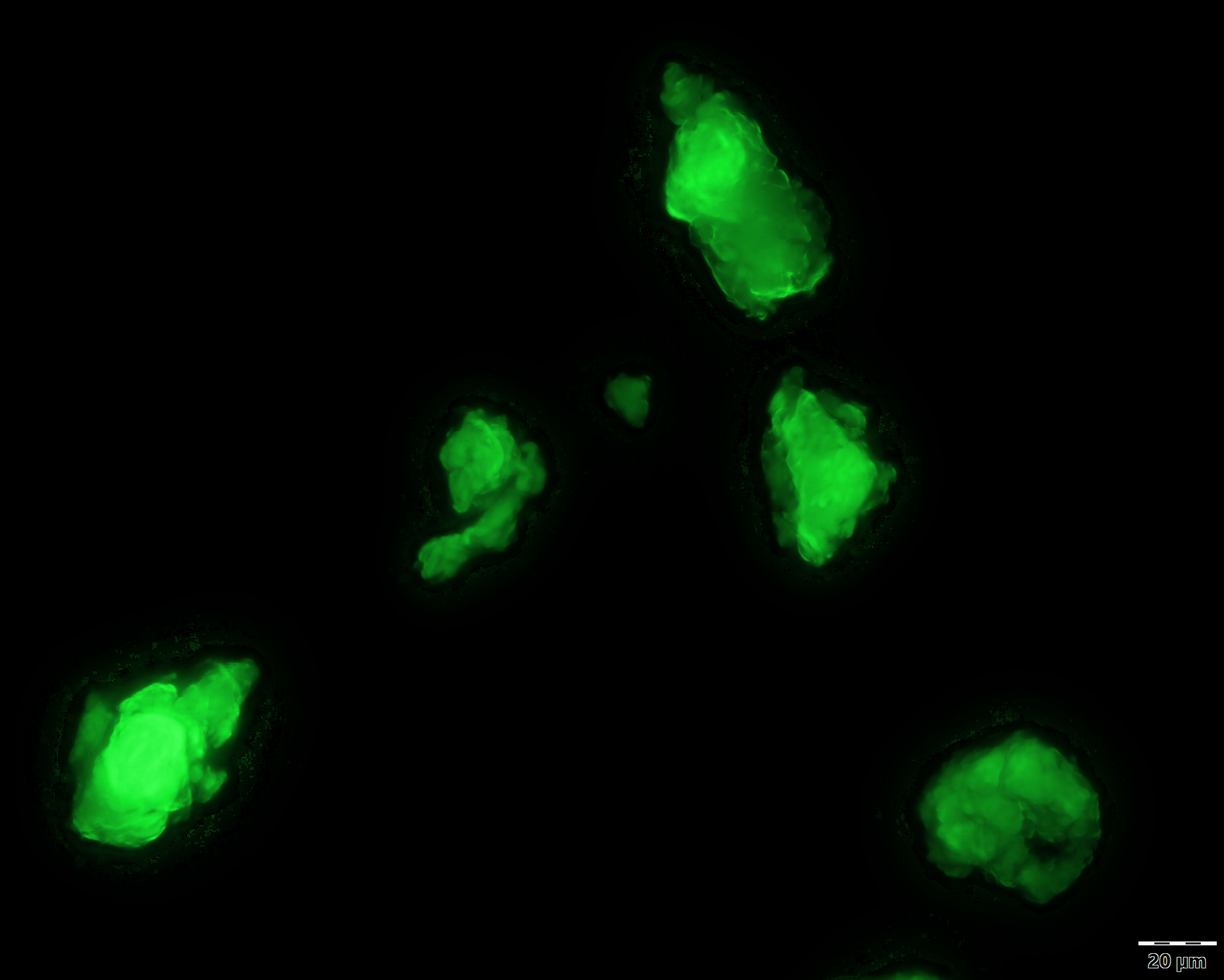

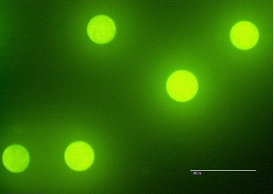

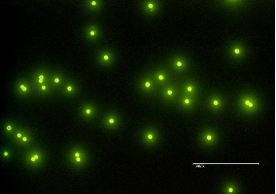

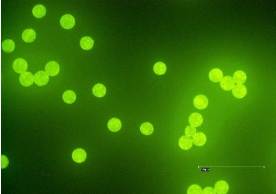


**a**

**b**

**c**

**d**

**Fig. S1** Fluorescent microscopic images of (a) 75–90 µm and (b) 38–45 µm PE beads, (c) 10 µm PS beads and (d) <63 µm PVC fragments used in the ingestion and egestion studies


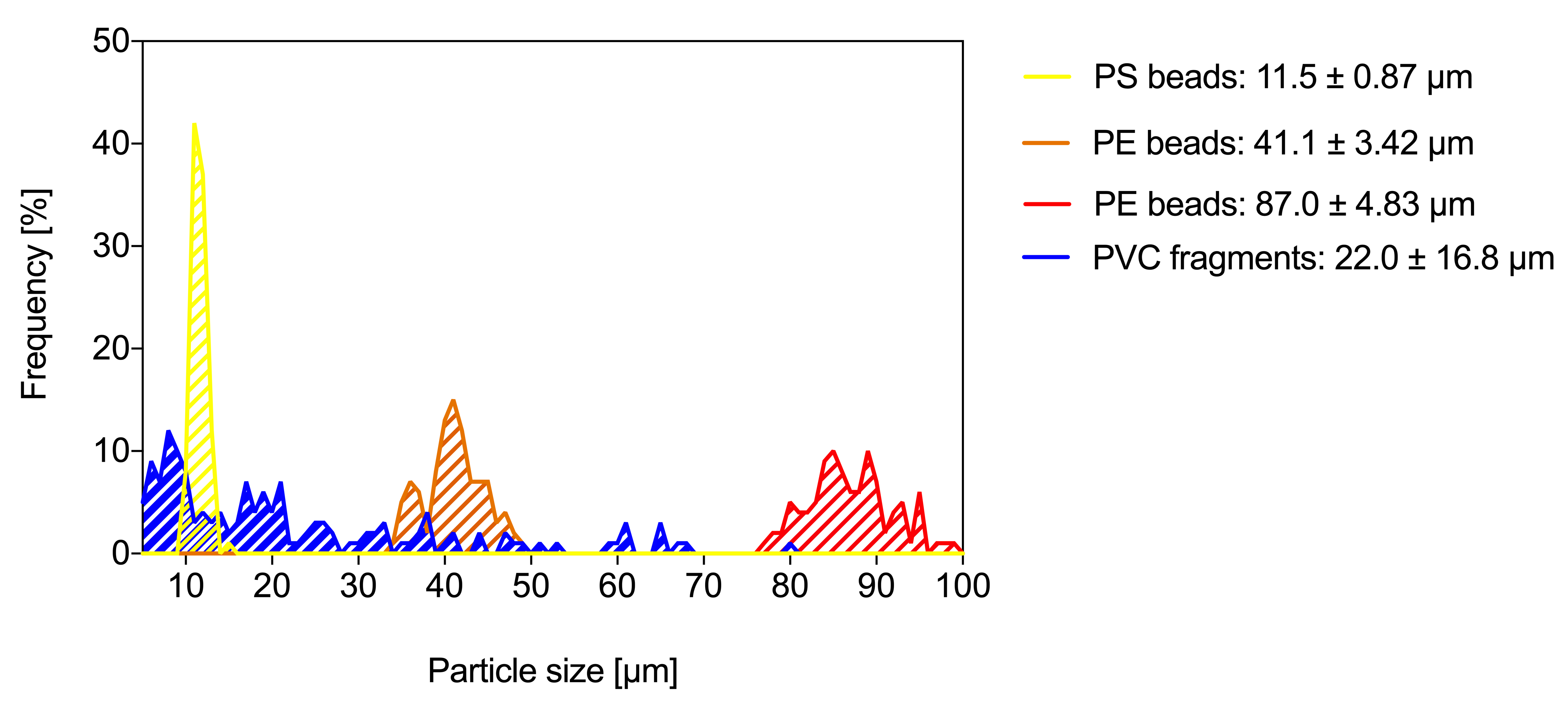


**Fig. S2** Size distributions and mean sizes (± SD) of beads and fragments used in the ingestion and egestion studies. *n* = 100 beads and *n* = 150 fragments (PVC fragments that were smaller than 5 µm are not included).

**Fig. S3** Surface structure of PVC fragments by scanning electron microscopy (SEM)


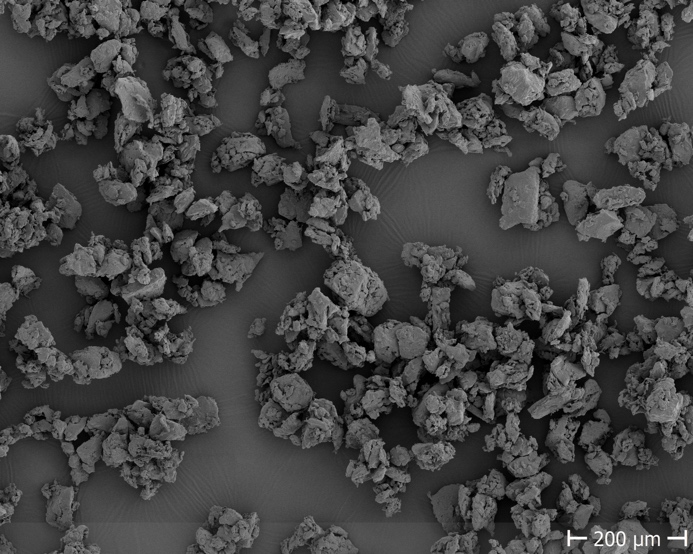

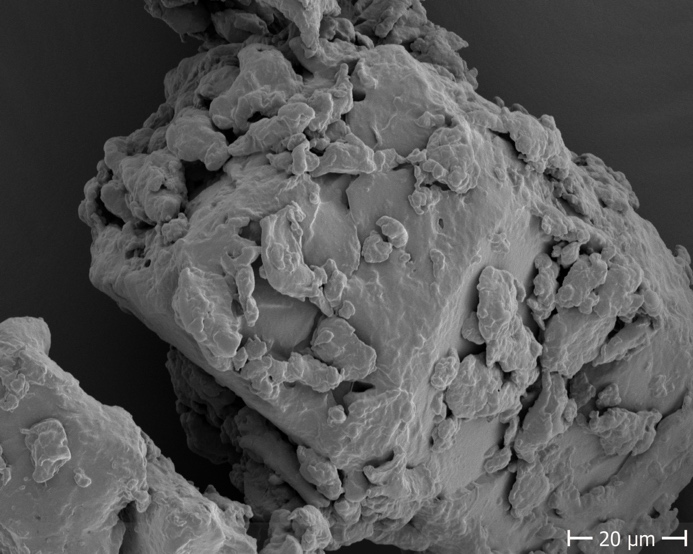


**Table S1** Volume or mass of beads and fragments used for the preparation of stock suspensions

| Sample | Beads | | | Fragments |
| --- | --- | --- | --- | --- |
|  | 10 µm PS | 38–45 µm PE | 75–90 µm PE | <63 µm PVC |
| Volume or mass | 110 µL L^-1^ | 91.9 and 414 mg L^-1^ | 407 mg L^-1^ | 20, 100 and 200 mg L^-1^ |

To prepare stock suspensions, the volume or mass of particles listed in Table S1 were suspended in ultrapure water (beads) or medium (fragments). Thereof, aliquots were taken, filtered and the retained particles counted using the microscope to derive the volumes corresponding to the test concentrations (procedure described in the main part). As an example, we prepared three filters and derived on average 13 ± 3 fragments for the lowest MP concentration (i.e., 20 MPs L^-1^ or 10 MPs 500 mL^-1^). Therefore, nominal and actual concentrations were not exactly but nearly the same. Similar observations were made for the other concentrations.


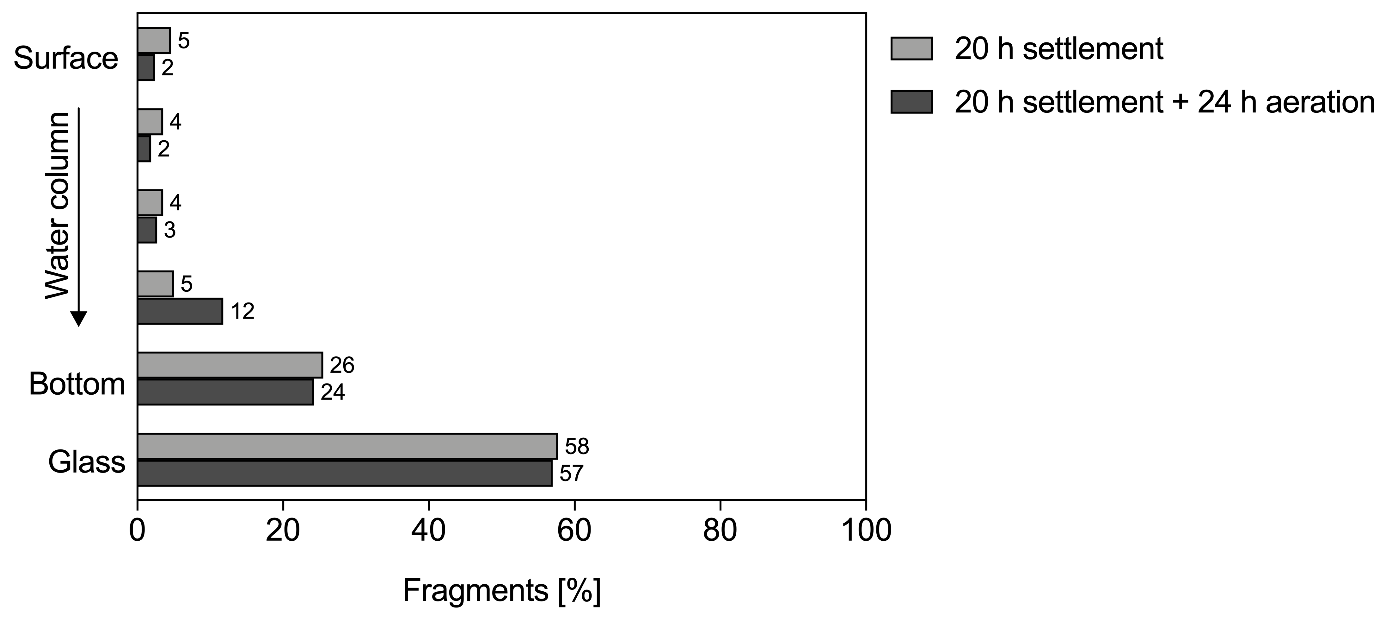


**Fig. S4** Fragment distribution after 20 h of settlement (*n* = 1) and additional 24 h of aeration (*n* = 1) in the test vessels containing 500 mL medium and spiked fragments. The medium was decanted in 100 mL steps and analyzed for fragments. 20 h settlement = 819 analyzed particles, 20 h settlement and 24 h aeration = 586 analyzed particles

After 20 h of settlement and 24 h of aeration, the contents of two beakers with 500 mL medium each containing 53 µL of the 100 mg L^-1^ (Table S1) fragment stock suspension, were decanted in 100 mL steps. Subsequently, the beakers were carefully rinsed with ultrapure water to account for particles attached to the glass in general and to the bottom of the glass. All aliquots and the rinsed water were analyzed for PVC fragments as described in the body of the text (see section 2.2). The majority of particles was observed in the bottom 100 mL of the water column and the glass (Fig. S3). Additional 24 h aeration did not resuspend the settled fragments.

**Table S2** Water parameters (mean ± SD) after the ingestion and egestion studies with *Neocaridina palmata*

| Experiment | Ingestion study | | Egestion study | | Mean |
| --- | --- | --- | --- | --- | --- |
|  | Beads | Fragments | Beads | Fragments |  |
| pH | 6.86 ± 0.02 | 7.23 ± 0.11 | 7.62 ± 0.19 | 7.46 ± 0.06 | 7.29 ± 0.30 |
| Conductivity [µS cm^-1^] | 314 ± 1.34 | 449 ± 3.82 | 359 ± 5.94 | 453 ± 0.47 | 394 ± 58.9 |
| Oxygen [mg L^-1^] | 8.89 ± 0.02 | 8.74 ± 0.23 | 8.92 ± 0.03 | 8.09 ± 0.02 | 8.66 ± 0.29 |
| Temperature [°C] | 23.2 ± 0.10 | 22.2 ± 0.78 | n.m. | 24.6 ± 0.05 | 23.3 ± 1.01 |

n.m. not measured

**Table S3** Sex ratio [%] and body length [mm] (mean ± SD) of *Neocaridina palmata* individuals

| Experiment | | Ingestion study | | Egestion study | | Mean |
| --- | --- | --- | --- | --- | --- | --- |
|  |  | Beads | Fragments | Beads | Fragments |  |
| Sex ratio [%] | Males | 62 | 44 | 65 | 61 | 58 |
|  | Females | 38 | 56 | 35 | 39 | 42 |
| Body length [mm] | Males | 11.7 ± 1.80 | 13.0 ± 0.96 | 11.9 ± 0.99 | 12.7 ± 0.96 | 12.3 ± 1.37 |
|  | Females | 12.8 ± 1.63 | 14.0 ± 1.02 | 12.7 ± 0.99 | 13.7 ± 1.26 | 13.3 ± 1.34 |
|  | Total | 12.1 ± 1.82 | 13.6 ± 1.13 | 12.2 ± 1.06 | 13.1 ± 1.19 | 12.7 ± 1.48 |

The wet weight was only assessed for the individuals exposed to fragments (i.e., the fragment ingestion study with and without food as well as the fragment excretion study) and not for the individuals exposed to beads. Hence, we did not include this parameter in the main part. The weight of the shrimps exposed to fragments was on average 56.5 ± 13.6 mg.

**Table S4** Mean number of detected beads and fragments (± SD) per treatment in the ingestion and egestion studies with *Neocaridina palmata.* Beads data are corrected for particles detected in the corresponding negative and solvent controls (NC and SC), which served as blanks. Fragment data are corrected for corresponding airborne and food blanks

| Study | Particle scenario | | Beads | Fragments | Fragments with food |
| --- | --- | --- | --- | --- | --- |
| Ingestion | SC | | 0 | ^-^ | ^-^ |
|  | NC | | 0 | 0 | 5.88 ± 4.42 |
|  | 20 particles L^-1^ | | 0.63 ± 0.74 | 6.13 ± 4.26 | 4.75 ± 3.69 |
|  | 200 particles L^-1^ | | 5.00 ± 5.29 | 12.8 ± 7.91 | 13.9 ± 14.3 |
|  | 2,000 particles L^-1^ | | 36.1 ± 37.2 | 47.4 ± 49.6 | 18.5 ± 27.8 |
|  | 20,000 particles L^-1^ | | 64.6 ± 40.2 | 204 ± 395 | 102 ± 170 |
| Egestion | NC | Lysate | 0 | n.m. |  |
|  |  | Excretions | 2.13 ± 1.36 | n.m. |  |
|  | 0 h | Lysate | 243 ± 148 | 371 ± 541 |  |
|  | 4 h | Lysate | 123 ± 338 | 14.0 ± 22.4 |  |
|  |  | Excretions | 143 ± 123 | 65.1 ± 31.7 |  |

n.m. not measured

For the fragment ingestion study, we detected 1.00 ± 1.41 (*n* = 3) PVC-like particles that were airborne. In the egestion study, no airborne PVC-like particles were detected, but the food source introduced 1.63 ± 1.91 (*n* = 8) PVC-like particles. This food blank included 8 replicates of only 500 mL ultrapure water and 10 mg food, which was aerated for 4 h and then filtrated to account for PVC-like fragments introduced by the food source. This food blank was used for the egestion study with fragments since food was added for the shrimps to facilitate excretion of previously ingested particles. The food blank was not used for the fragment ingestion study with food, since the respective negative control already included food and thus, accounted for the introduction of PVC-like fragments by the food source.
